# To test or not to test? A new behavioral epidemiology framework for COVID-19

**DOI:** 10.1371/journal.pone.0309423

**Published:** 2024-12-17

**Authors:** Jayanta Sarkar

**Affiliations:** School of Economics and Finance, Queensland University of Technology, Brisbane, QLD, Australia; Lahore School of Economics, PAKISTAN

## Abstract

Evidence from clinical research suggests that in the first two waves of COVID-19, the virus spread rapidly through a large number of undocumented asymptomatic infections. These ‘silent’ infections camouflaged the actual incidence of the disease, leading to downward biases in the rates of transmission, disease prevalence, and fatality. These, in turn, had implications for how people and policymakers responded to changing infection prevalence. This paper posits that in the early stages of the COVID-19 pandemic, a considerable number of SARS-CoV-2 infections spread through asymptomatic infected individuals who lacked economic incentives to test and isolate adequately. The decision to undertake testing and the subsequent possibility of isolation entails a calculus of benefits and costs for an individual. Given that the perceived net benefit of such actions is correlated with the observed risk of infection, the likelihood of an asymptomatic individual choosing to undergo testing increases with the existing infection prevalence rate. This behavior, in turn, influenced disease transmission and mortality dynamics. This study presents an analytical framework that integrates prevalence-dependent testing behavior into a traditional epidemiological model. The model’s predictions provide critical policy insights. It reveals that failing to account for testing and isolation behavior results in underestimation of the infection propagation and fatality rates when reported disease prevalence is low, thereby, skewing the containment strategies in the initial and late stages of a pandemic. The findings underscore the necessity of enhancing testing capacity as a crucial countermeasure for future contagions like COVID-19.

## 1. Introduction

Economists have long sought to understand how individuals respond and adapt to disease threats and how these adaptive behaviors influence disease transmission and mitigation policies. The COVID-19 pandemic has presented a rare opportunity to examine these disease-behavior interactions that emerged due to a crucial aspect of disease transmission–’silent’ infections. Silent infections are propagated by asymptomatic individuals who, despite being infectious, remain without noticeable disease symptoms throughout the course of infection. Clinical research using seroprevalence data has revealed that hidden asymptomatic infections accounted for a significant proportion of COVID-19 cases in the initial phase of the pandemic and were a major source of SARS-CoV-2 dissemination [1–3]. Available estimates of prevalence of asymptomatic SARS-CoV-2 infections vary widely across different populations, with figures ranging from 1.2–91.9% of all recorded infections [4–6]. These undetected asymptomatic infections led to substantial and yet unquantified differences between observed and actual disease prevalence. The resulting uncertainty posed significant challenges to COVID-19 mitigation efforts that relied on symptom-based testing strategies.

The success of these strategies largely depends on individual willingness to test, and self-isolate for a period if testing positive. In most settings diagnostic testing is an individual choice based on evaluation of private costs and benefits. While testing and subsequent self-isolation by an infectious person can potentially protect the health of other household members and close contacts and prevent further spread of infection, self-isolation can negatively impact employment and income, mobility, and social activities. Indeed, the requirement for self-isolation following a positive test result, and the consequent loss of income, were identified as key barriers to testing in the US and elsewhere [7–10]. Unsurprisingly, non-compliance and refusal to undergo testing emerged as stubborn public health issues in some countries [11]. Non-compliance with testing protocols is likely to be more prevalent among asymptomatic individuals, as they may underestimate the necessity of testing in absence of discernible symptoms. For policymakers, compliance with testing and isolation protocols is essential for accurate disease risk assessment. However, non-compliance represents a significant behavioral challenge to the efficacy of infection control measures. In the pandemic’s early stages, this diagnostic challenge obscured the true prevalence of SARS-CoV-2 infections despite extensive tracking and testing efforts worldwide.

This paper aims to provide a better understanding of role of behavior in the dynamics of viral transmission during the early, pre-vaccination stages of the COVID-19 pandemic. It explores how individual mitigating actions may have influenced the infection curve’s initial flattening and subsequent elongation. By examining the interplay between behavior and disease progression, the paper aims to enhance policy strategies for managing future pandemics. Central to this analysis is the integration of adaptive testing and isolation behaviors among asymptomatic carriers (henceforth, the asymptomatics) into a conventional epidemiological model, offering insights into the pandemic’s trajectory.

In this framework, the economic choice to undertake testing and isolation (henceforth, T&I) entails private benefits from protecting self and others. T&I also entails private cost associated with a protracted self-isolation period if tested positive, that is not covered by insurance. So long as the absence of physical disease symptoms reduces the perceived risk of being infected, the asymptomatics will have lower incentives for T&I compared to those with palpable symptoms (henceforth, the symptomatics), given everything else. This perceived risk, and consequently the motivation for T&I increase in response to observed disease threats–such as, reported infection prevalence. Assuming self-isolation always follows a positive test result, testing and isolation are treated as a unified decision. An alternative model, presented in S1 File, considers self-isolation as a separate compartment with the possibility of imperfect compliance.

The behavioral-epidemiological model presented here provides a theoretical foundation for the hidden infections and predicts significantly higher equilibrium transmission rate and mortality compared to the standard models. One of the key insights is that the peak of infection is self-limiting because preventive T&I behavior is activated in response to rise in observed prevalence. On the downside, infections are also predicted to linger at the tails when T&I falls with decreasing prevalence, making complete eradication of the virus extremely difficult. The simulated model successfully replicates the fast-changing daily data from Italy in the first wave of the pandemic, which establishes the external validity of the model.

This research contributes to the expansive economic-epidemiological literature in two ways. First, it incorporates endogenous behavioral responses within an epidemiological framework, which remain largely unexplored, except in a handful of studies on the use of prophylactics [12–16]. Endogenous behavior around diagnosis and transmission, rather than behavior around exposure avoidance, has not been explored so far. Second, it contributes to the COVID-19 literature by underscoring the critical role of asymptomatic transmissions in influencing the disease indicators and policy. Many recent COVID-19 studies modified standard models to reflect variable contact rates among different groups [17], overlooking behaviors like test avoidance associated with asymptomatic infections—despite increasing evidence of such phenomena. Most studies on economy-COVID-19 interaction use different variations of disease transmission probability modified to capture social interaction and policy response decisions [18–20]. T&I as a relevant endogenous behavior and its implications for disease transmission and severity has not been analyzed in the literature. By highlighting the interplay between behavior and viral propagation, this paper narrows the gap between the economics and epidemiology disciplines.

The implications of endogenous T&I are significant for the success of future disease management. First, by failing to recognize the asymptomatic carriers, symptom-driven testing strategies underestimate the true magnitude of community transmission and bias the severity of COVID-19. Second, an underestimation of the infection risk may skew the implementation and extent of containment measures, thereby disrupting the intricate equilibrium between managing the disease’s impact and mitigating its economic repercussions throughout the pandemic. While the spread and virulence of COVID-19 infections are much diminished at the time of publication, knowledge of true infection prevalence is remains important given the emergence of novel SARS-CoV-2 variants and the cumulative immunity conferred by previous infections and vaccinations, which may contribute to an increased incidence of asymptomatic infections [21, 22]. Third, an underestimation of the prevalence rate distorts the herd immunity target and the policies targeted to achieving herd immunity and subsequently ending preventive social and travel restrictions. In this scenario, increasing testing capacity, such as the development of accurate and rapid self-test kits, can be an effective health policy measure for the containment of the virus.

The rest of the paper is structured as follows. Section 2 provides a brief overview of the Susceptible-Latent-Infected-Recovered (SLIR) model–a widely used compartmental epidemiological model. Section 3 outlines the economic rationale for prevalence-dependent T&I behavior among the asymptomatics and analyses the equilibrium outcomes of a standard model that incorporates this behavioral foundation. The policy significance of the results is discussed in Section 4, while the results of a simulated model, highlighting the effectiveness of two alternative public policy measures, are elaborated in Section 5 along with the replication of Italian data. Section 6 concludes.

## 2. The standard SLIR model

We begin with the pioneering model of Kermack and McKendrick [23] that provides a simple background framework for a compartmental model. Assume that at a given time, an individual can belong to only one of the four compartments–‘Susceptible’ (at risk of infection), ‘Latent’ or exposed (infected, but clinical symptoms are yet to manifest), ‘Infectious’ (transmits the disease), or ‘Recovered’ with life-long immunity. Individuals start as susceptibles and transition through these sequential phases, ending their infection lifecycle as recovered or ‘removed’ if the disease is fatal. Let at any time *t* the number of people in susceptible, exposed, infectious, and recovered/removed compartments be denoted by *S*(*t*), *L*(*t*), *I*(*t*), *R*(*t*), respectively. The transmission of the disease follows the principle of mass action or bilinear incidence, implying that *βS*(*t*)*I*(*t*) number of susceptible individuals move from *S* to *L* group per period, where *β* is the infection transmission rate. Let *κ*>0 be the rate at which each exposed person with latent infection transitions to the infectious stage, which implies an average latency period of 1/*κ* days. Also, *ω*>0 denotes the rate of recovery, indicating a mean infectious period of 1/*ω*. In a closed system with non-fatal disease, the population size remains constant and is given by *N* = *S*(*t*)+*L*(*t*)+*I*(*t*)+*R*(*t*). The system of ordinary differential equations for the SLIR model is given by:

$$S^{\prime }=-\beta I(t)S(t)$$ (1A)

$$L^{\prime }=\beta I(t)S(t)-\kappa L(t)$$ (1B)

$$I^{\prime }=\kappa L(t)-\omega I(t)$$ (1C)

$$R^{\prime }=\omega I(t)$$ (1D)

To move to a disease-free equilibrium (DFE), the number of infectious must decline with time. Evaluating the condition *L*′+*I*′<0 at *t* = 0, would yield *βS*(0)<*γ*, or

$$R_{0}\equiv \beta S_{0}/\gamma$$ (2)

*R*_0_ is a threshold quantity, known as the *basic reproduction number*, that determines the average number of susceptible individuals that a newly introduced infected person would infect in a fully susceptible population of size *S*(0) = *S*_0_. If *R*_0_<1, *I*(*t*) decreases monotonically to zero; whereas if *R*_0_>1, this number first increases, reaches a peak and declines to zero; thus *R*_0_ = 1 acts as a sharp threshold between the disease dying out or causing an epidemic. The model can generate some key indicators, such as the maximum infection rate, the fraction of population infected (attack ratio), etc. that are useful for disease containment policy.

Before we proceed, it is important to note that this is a theoretical and observational study. The relevant Human Research Ethics Committee has confirmed that no ethical approval is required.

## 3. Asymptomatic COVID-19 infections and rational disease dynamics

The standard SLIR model assumes that infection status is always revealed through observable symptoms following a pre-symptomatic latency period. However, in contrast to the preceding SARS-CoV and MERS-CoV outbreaks, a unique feature of SARS-CoV-2 transmission has been that a significant proportion of infected individuals remain asymptomatic throughout their infection lifecycle [3].

The key idea explored here is that in the absence of physical symptoms, the asymptomatics discount the likelihood of contracting the virus and therefore have lower incentive to undertake preventive steps, such as volunteering to T&I. The implications of testing positive may deter them from testing. If tested positive, they will be unable to leave their homes, return to work, and spend time with their families for a considerable time. These private costs are uninsurable, yet they can be circumvented by opting out of testing. Evidence suggests that in the first wave of COVID-19, infected people with no or mild symptoms failed to present at healthcare facilities or appear for testing [24, 25]. Some refused to test, even when symptomatic [26]. The lack of incentive for T&I may have been more widespread among the asymptomatics, that resulted in many undetected transmissions. Indeed, scientific evidence strongly suggests that these hidden infections were a dominant force behind the surge of infections of SARS-CoV-2 [1–3].

Nonetheless, maladaptive behavior can adapt to infection prevalence, as explored in the literature. This research indicates that the demand for preventive measures, particularly vaccination, increases with rising infection rates [12, 27, 28]. Sarkar [29] provides cross-country evidence that COVID-19 vaccine demand rises in response to infection prevalence and severity. Similar behavioral response is evidenced during the pre-vaccine period of COVID-19 pandemic. Results from a recent randomized controlled trial in Mozambique demonstrate that adherence to preventive policies, such as social distancing, responds positively to perceived rates of infection [30]. If diagnostic demand rises with prevalence rate, the test positivity rate (fraction of all COVID-19 tests that return a positive result) should be positively correlated with infection rate. Indeed, seroprevalence data from the United States showed demand for testing was strongly predicted by reported prevalence rate and test positivity rate [31]. Cross-country data suggest that voluntary testing responds positively to infection prevalence [32]. Hence in our model private demand for T&I among the asymptomatics depends upon the current infection prevalence rate.

### 3.1 An augmented SLIR model with rational preventive behavior

Let us set the background by providing a theoretical argument for prevalence dependence of T&I behavior. Here, the infected individuals with no or little symptoms are unaware of their infectivity and the threat they pose to others. Hence, their T&I decision is informed by the perceived probability of being exposed to the virus and the associated costs and benefits. Given the transmission rate *β*∈(0,1), if the probability of a random contact occurring between a susceptible and an infected (following the principle of *mass action*) be given by the prevalence rate *i*≡*I*/*N*, the probability of an infected person infecting a susceptible at any time *t* is given by *p*_*t*_ = *βi*_*t*_. This formulation is similar to Geoffard and Philipson [27], Philipson [12], and Jones et al. [33], where the probability of contracting an infectious disease by a susceptible is proportional to the existing prevalence rate.

In our model, individuals are either non-infected, symptomatics, or asymptomatics. Individuals derive utility or satisfaction from their own health status. Let $u_{t}^{ni},\;u_{t}^{is},\;u_{t}^{ia}$ denote the levels of satisfaction or utilities of a non-infected, symptomatic, and asymptomatic, respectively at date *t*. Disease symptoms, however mild, reduce utility from health so that $u_{t}^{ni}\geq u_{t}^{ia}>u_{t}^{is}$. In addition, individuals derive utility from the health of the susceptible people they live, work, and socialize with (close contacts). Let the collective utility from the health status of one’s close contacts be given by $u_{t}^{o}$, which is weighted by a factor *φ*>0 that reflects its relative importance to the individual. An infected individual transmits the virus to a susceptible close contact with probability *p*_*t*_, which reduces $u_{t}^{o}$ by a factor *z*>0. Assume that individuals incur a positive cost *c*_*T*_>0 if they want to get tested. *c*_*T*_ includes all cost of testing, such as transportation cost to the testing centers, cost of purchase of home test kits, time-cost involved in the testing process, psychic and time cost of locating PCR testing centers if not available in the neighborhood, and psychic and physical discomfort from the test procedure, etc. Conditional on testing positive, an infected must self-isolate for a considerable period (generally 14 days), and in the process incurs a cost: $c_{q}^{is}$ and $c_{q}^{ia}$ for the symptomatics and the asymptomatics, respectively. These costs encompass the foregone social and economic opportunities, as well as the significant psychological costs of isolation, particularly as the pandemic-induced economic and social barriers are lifted. Because the psychic cost of isolation is likely to be higher for the asymptomatics, we expect$c_{q}^{ia}>c_{q}^{is}>0$.

The decision to test is analyzed in a simple two-period model framework. Two infected individuals–one representing symptomatic and the other asymptomatic–seek to maximize their expected utility over the two periods by choosing whether to undergo testing. In period *t*, each individual is aware of possible exposure to the virus, considering the option of testing in the post-latency period *t*+1. To do so, each compares the expected utilities corresponding to the outcomes with and without testing. The symptomatic person, whose current utility is $u_{t}^{is}$ weighs the ex-ante utilities from testing vis-à-vis not testing, taking the probability of being infected, *p*_*t*_ as given. Let *ϑ*>0 be the probability of death for an infected and *α*>0 be the temporal discount factor, regardless of symptom status. All future utilities are weighted by the effective discount factor *α*(1−*ϑ*). If she decides not to test and survives the next period, her future utility from own health is $p_{t}u_{t+1}^{is}+(1-p_{t})u_{t+1}^{ni}$ and that from the health of close contacts is $\varphi p_{t}(u_{t+1}^{o}-z)+\varphi (1-p_{t})u_{t+1}^{o}$. If she undergoes testing and isolation, she incurs a current cost *c*_*T*_, and receives a future utility of $p_{t}(u_{t+1}^{is}-c_{q}^{is})$. Also, because self-isolation follows a positive test result, $u_{t+1}^{o}$ obtains with certainty. In this set up everything is symmetric for the asymptomatic individual.

Let the expected utility of the symptomatic individual be given by:

$$EU_{t+1}^{is}=max\left\{\begin{array}{c} {}[u_{t}^{is}+\alpha (1-\vartheta )\{p_{t}u_{t+1}^{is}+\varphi p_{t}{(u}_{t+1}^{o}-z)+(1-p_{t})u_{t+1}^{ni}+\varphi (1-p_{t})u_{t+1}^{o}\}];\\ \left[u_{t}^{is}-c_{T}+\alpha (1-\vartheta )\{p_{t}(u_{t+1}^{is}-c_{q}^{is})+(1-p_{t})u_{t+1}^{ni}+\varphi u_{t+1}^{o}\}\right] \end{array}\right\}$$ (3)

Similarly, the expected utility of the asymptomatic individual is:

$$EU_{t+1}^{ia}=max\left\{\begin{array}{c} {}[u_{t}^{ia}+\alpha (1-\vartheta )\{p_{t}u_{t+1}^{ia}+\varphi p_{t}{(u}_{t+1}^{o}-z)+(1-p_{t})u_{t+1}^{ni}+\varphi (1-p_{t})u_{t+1}^{o}\}];\\ \left[u_{t}^{ia}-c_{T}+\alpha (1-\vartheta )\{p_{t}(u_{t+1}^{ia}-c_{q}^{ia})+(1-p_{t})u_{t+1}^{ni}+\varphi u_{t+1}^{o}\}\right] \end{array}\right\}$$ (4)

It can be shown that there exist uniquely continuous expected utility functions that satisfy Eqs (3) and (4). From Eq (3), a symptomatic individual will choose to test *if*:

$$u_{t}^{is}+\alpha (1-\vartheta )[p_{t}u_{t+1}^{is}+p_{t}\varphi {(u}_{t+1}^{o}-z)+(1-p_{t}){(u}_{t+1}^{ni}+\varphi u_{t+1}^{o})]\leq u_{t}^{is}-c_{T}+\alpha (1-\vartheta )[p_{t}(u_{t+1}^{is}-c_{q}^{is})+(1-p_{t})u_{t+1}^{ni}+\varphi u_{t+1}^{o}].$$

This condition simplifies to yield a threshold probability of testing for the symptomatics $p^{*is}=\frac{c_{T}}{\alpha (1-\vartheta )(\varphi z-c_{q}^{is})}$, and for the asymptomatics, $p^{*ia}=\frac{c_{T}}{\alpha (1-\vartheta )(\varphi z-c_{q}^{ia})}$. Given *p* = *βi*, these quantities also reflect the critical disease prevalence rate that triggers T&I behavior. The threshold probabilities increase with cost of testing (*c*_*T*_) and isolation ($c_{q}^{ia}$) and decrease in the cost of health of close contacts (*z*). Thus, the higher the T&I-related costs and lower the level of importance one attaches to communal health, the less likely an infected individual will choose to test. Given $c_{q}^{ia}>c_{q}^{is}$, this threshold is higher for the asymptomatics, who are, therefore, expected to be less likely to test than the symptomatics, given everything else.

Distinct from the existing studies on prevalence-dependence, the perceived exposure probability and hence the probability of T&I is assumed to depend on the *reported* prevalence rate, which is based on the number of detected positive cases, *T*(*t*). In what follows, the probability of undertaking T&I by a symptomatic and an asymptomatic would be given by *p*^*s*^ and *p*^*a*^, respectively, where *p*^*a*^ is increasing in the infection prevalence rate.

**Fig 1:**
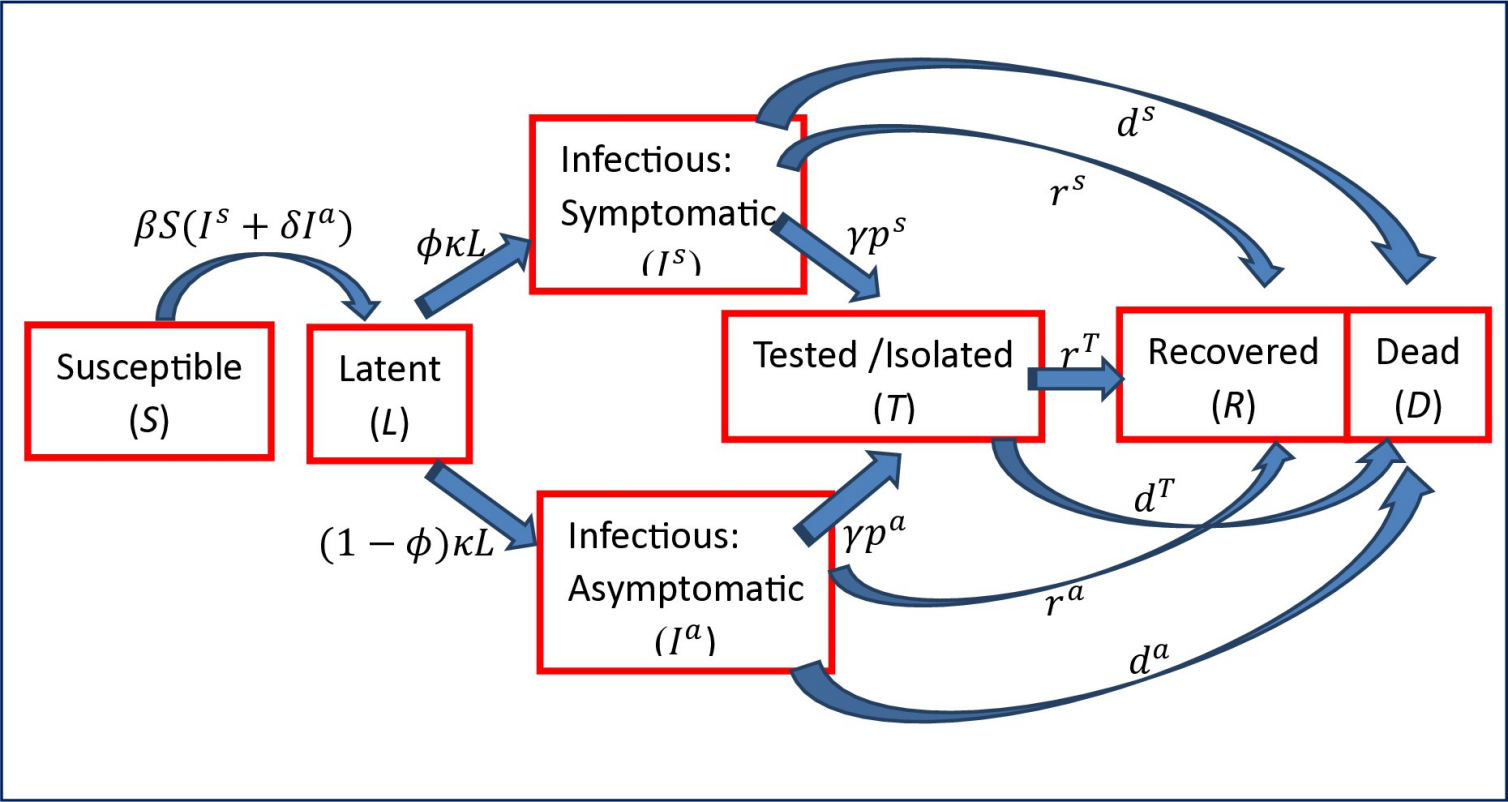
Disease transmission in the SLIITReD model.

To account for the behavioral features described above, I augment the canonical SLIR model by including a latent or gestation stage and a T&I stage preceding the Recovery state. The latent stage allows for some time for the state of infection to be revealed as symptomatic or asymptomatic. We assume that a fraction *ϕ* of the infected population becomes symptomatic. The T&I stage *T*(*t*) captures the part of the population who choose to test and isolate at time *t*. Thus, people in the *T* compartment are non-transmitter of the disease. The transition across health states in the proposed Susceptible-Latent-Infectious-Tested/Isolated-Recovered-Dead (SLIITReD) model can be represented in Fig 1.

Given that the information set for the observed epidemiological states is denoted by {*S*(*t*), *T*(*t*), *R*(*t*), *D*(*t*)}, the asymptomatic individuals’ beliefs that they have indeed been exposed to the virus would be proportional to the reported positive cases *T* as a fraction of people in these epidemiological states (either susceptible or known to have been infected). The probability that asymptomatic individuals believe they have been exposed, and therefore their likelihood of undergoing T&I at time *t*, is represented by: $p^{a}(t)=\mu T(t)/[S(t)+T(t)+R(t)+D(t)]$, where *μ*>0 is a sensitivity parameter.

Given $p^{a}(t)\leq 1,\mu \leq max\left[\{S(t)+R(t)+D(t)\right\}/T(t)]$. Note that $p^{a}(0)=\mu T_{0}/\left(S_{0}+T_{0}\right)$, where ${S(0)=S}_{0}>0,T(0)=T_{0}\geq 0$ and $\underset{t\to \infty }{\lim}p^{a}(t)=\underset{t\to \infty }{lim}T(t)=0$. Normalizing *N* = 1, the dynamic system the model is represented by the following ODEs:

$$S^{\prime }=-\beta \{I^{s}(t)+{\delta I}^{a}(t)\}S(t)$$ (5A)

$$L^{\prime }=\beta \{I^{s}(t)+{\delta I}^{a}(t)\}S(t)-\kappa L(t)$$ (5B)

$${I^{s}}^{\prime }=\phi \kappa L(t)-\{{\gamma p}^{s}+{r^{s}+d}^{s}\}I^{s}(t)$$ (5C)

$${I^{a}}^{\prime }=(1-\phi )\kappa L(t)-\{{\gamma p}^{a}(t)+{r^{a}+d}^{a}\}I^{a}(t)$$ (5D)

$$T^{\prime }={\gamma p^{s}I}^{s}(t)+{\gamma p}^{a}(t)I^{a}(t)-(r^{T}+d^{T})T(t)$$ (5E)

$$R^{\prime }=r^{s}I^{s}(t)+{r^{a}I}^{a}(t)+r^{T}T(t)$$ (5F)

$$D^{\prime }=d^{s}I^{s}(t)+{d^{a}I}^{a}(t)+d^{T}T(t)$$ (5G)

$$p^{a}(t)=\frac{\mu T(t)}{S(t)+T(t)+R(t)+D(t)}$$ (5H)

As in the mainstream epidemiological models, the incidence rate is assumed to be bilinear. The asymptomatics are assumed to carry a lower viral load and therefore have a lower infectivity denoted by *δ*<1. The average latency period is 1/*κ*; the death rate and recovery rate of the symptomatic infected are *d*^*s*^ and *r*^*s*^, while these values for the asymptomatics are *d*^*a*^ and *r*^*a*^, respectively. The recovery and death rates under isolation is *r*^*T*^ and *d*^*T*^, respectively.

Before proceeding it is useful to note that like all models, the current model also abstracts from some real-life complexities. For example, it excludes differing infectivity and virulence of different variants of SARS-CoV-2, demographic differences in susceptibility, infectivity, and morbidity, the implications of access to effective vaccines, testing prioritization for high-risk demographic groups, interaction with other health policies in place, quarantine requirements of close contacts, and many such issues. We do relax some of these assumptions to test the robustness of the model. For example, we show that the qualitative nature of the model remains unchanged when it (i) incorporates a distinct self-isolation compartment with imperfect compliance with self-isolation, and (ii) accounts for transient immunity after recovery. Indeed, many real-life intricacies can be included, but it will unnecessarily complicate the model without enhancing insights on the key issue of disease-behavior interaction. So, the model excludes many real-life considerations in favor of parsimony and clarity.

This system nests the standard SLIR model which can be recovered by setting *I*^*s*^(*t*) = *I*^*a*^(*t*) and *T*(*t*) = 0; *p*^*s*^ = *p*^*a*^ = 1; *r*^*s*^ = *r*^*a*^ = *r*; *d*^*s*^ = *d*^*a*^ = 0. As in the standard model this behavioral model describes diseases in which recovered individuals are immune to reinfection. Note that the Mass conservation property holds, because $S^{\prime }+L^{\prime }+{I^{s}}^{\prime }+{I^{a}}^{\prime }+T^{\prime }+R^{\prime }+D^{\prime }=0$. Hence, $S(t)+L(t)+I^{s}(t)+I^{a}(t)+T(t)+R(t)+D(t)=N=1$. The system with seven differential equations is positive as all state variables take non-negative values for *t*≥0, given non-negative initial conditions. Note that *R*(*t*) and *D*(*t*) are cumulative variables that depend only on the other ones and their own initial conditions.

Given the set of initial conditions $\left\{S(0),L(0),I^{s}(0),I^{a}(0),T(0),R(0),D(0)\right\}$ and *p*^*a*^(0)≥0, the variables converge to the *DFE* given by: $\left(S^{*},L^{*},{I^{s}}^{*},{I^{a}}^{*},T^{*}R^{*},D^{*})=\{\bar{S},0,0,0,0,\bar{R},\bar{D}\right\}$, with $\bar{S}+\bar{R}+\bar{D}=1$. This set represents all possible equilibria. The basic reproduction number is derived in S1 File using the new-generation approach as the maximum element in the spectrum of the next-generation matrix. The behavioral *R*_0_ is given by:

$$R_{0}^{b}=\frac{\beta S_{0}\{\delta (1-\phi )(\gamma p^{s}+r^{s}+d^{s})+\phi (\gamma {{p^{a}(0)+r}^{a}+d}^{a})\}}{\left({\gamma p}^{s}+r^{s}+d^{s})(\gamma p^{a}(0)+{r^{a}+d}^{a}\right)}=\beta S_{0}\left(\underset{Symptomatics}{\underset{︸}{\frac{\phi }{{\gamma p}^{s}+r^{s}+d^{s}}}}+\underset{Asymptomatics}{\underset{︸}{\frac{\delta (1-\phi )}{{{\gamma p^{a}(0)+r}^{a}+d}^{a}}}}\right)$$ (6)

Thus, $R_{0}^{b}$ neatly decomposes into two parts: $R_{0}^{s}\equiv \frac{\beta \phi S_{0}}{{\gamma p}^{s}+r^{s}+d^{s}}$ and $R_{0}^{a}\equiv \frac{\beta \delta (1-\phi )S_{0}}{{\gamma p^{a}(0)+r}^{a}+d^{a}}$, such that $R_{0}^{b}=R_{0}^{s}+R_{0}^{a}$. Standard predictions that fully discount the existence of asymptomatic infections, would severely underestimate *R*_0_ by omitting the second term in Eq (6). Furthermore, given $dR_{0}^{b}/dp^{a}(0)<0$, the standard models that do recognize asymptomatic infections but assume exogenous and uniform T&I behavior across the infective groups *p*^*a*^(0)≈*p*^*s*^, would also underestimate *R*_0_.

As expected, more effective epidemic containment measures, such as social-distancing and lockdown, that reduce the magnitude of *β* reduce $R_{0}^{b}$. Note that *βδS*_0_ in the numerator of $R_{0}^{a}$ are the newly exposed individuals generated by one asymptomatic infectious individual per unit of time in an entirely susceptible population. A fraction 1−*ϕ* of them progress from the exposed stage to the infectious stage, each of whom remains infectious in the community for a period of 1/(*γp*^*a*^(0)+*r*^*a*^+*d*^*a*^). Therefore, $R_{0}^{a}$ denotes the number of secondary infections that one asymptomatic will produce in an entirely susceptible population during his/her lifespan. $R_{0}^{s}$ denotes the counterpart for a symptomatic.

As always, if $R_{0}^{b}<1$ there will be no epidemic. An epidemic will ensue in a virgin population if $R_{0}^{b}>1.$ In the latter case, infection cases will increase, but eventually fall because *S* falls monotonically (*S*’<0 for all *t*). Denoting $\underset{t\to \infty }{\lim}L(t)=L_{\infty }=0$ and $\underset{t\to \infty }{\lim}S(t)=S_{\infty }$, the final size relation is given by: $S_{0}(lnS_{0}-lnS_{\infty })=R_{0}^{b}(S_{0}-S_{\infty })+L_{0}R_{0}^{b}$. The incidence rate among the symptomatics is *ϕ*(*S*_0_−*S*_∞_)/*S*_0_ and that among the asymptomatics is (1−*ϕ*)(*S*_0_−*S*_∞_)/*S*_0_.

Once the outbreak is underway, the interaction between testing behavior and prevalence makes the reproduction number time varying. The e*ffective* reproduction number at any time *t*>0, is given by:

$$R_{t}^{b}=\beta S(t)\left(\frac{\phi }{\gamma p^{s}+r^{s}+d^{s}}+\frac{\delta (1-\phi )}{\gamma \mu \{T(t)/ℵ(t)\}+{r^{a}+d}^{a}}\right)$$ (7)

where, ℵ(t)≡S(t)+T(t)+R(t)+D(t). A higher testing rate (*γ*) and faster rates of transition from the infectious category to recovery and death decrease $R_{t}^{b}$. The innovation in this behavioral model is that the effective reproduction number depends on the endogenous probability *p*^*a*^ through the quantity *T*(*t*)/ℵ(*t*). Specifically, the magnitude of $R_{t}^{b}$ is decreasing in *T*(*t*)/ℵ(*t*)–meaning, given everything else, the higher this ratio, the higher is the probability of T&I for an asymptomatic, the faster is the rate at which the infectious are removed from future contact possibilities with the susceptibles. Therefore, a greater sensitivity of testing behavior to infection prevalence among the asymptomatics (*μ*) decreases both $R_{0}^{b}$ and $R_{t}^{b}$. The effective reproduction number is derived in S1 File.

Given the critical role of adaptive T&I behavior, it is useful to know the magnitude of *p*^*a*^ that determines the threshold around which infections change course. This threshold can be derived by solving the value of *p*^*a*^ at which the infection rate is maximum–that is when *I*^*s*′^+*I*^*a*′^ = 0, which obtains when the rate at which infectious people enter, *κ*, equals the rate at which they are removed either by isolation, recovery or death $\phi (\gamma p^{s}+r^{s}+d^{s})+(1-\phi )(p^{a}(t)+{r^{a}+d}^{a})$. This condition solves ${\hat{p}}^{a}={\left[\gamma (1-\phi )\right]}^{-1}[\kappa -\phi (\gamma p^{s}+r^{s}+d^{s})-(1-\phi )({r^{a}+d}^{a})]$. Therefore, the infection rate rises as long as $p^{a}(t)<{\hat{p}}^{a}$, and falls otherwise. The magnitude of ${\hat{p}}^{a}$ can be inferred from data.

The qualitative results are robust to more general specifications of *p*^*a*^(*t*) in Eq (5H) that includes disease mortality, such as $p^{a}(t)=\frac{\mu_{1}T(t)+\mu_{2}D(t)}{\rho_{1}S(t)+\rho_{2}T(t)+\rho_{3}R(t)+\rho_{4}D(t)}$ with *μ*_1_, *ρ*_1_>0 and *μ*_2_, *ρ*_2_, *ρ*_3_, *ρ*_4_≥0. The current specification, however, allows for a cleaner interpretation.

## 4. Significance for public health policy

The behavioral model has serious implications for the reliability of SARS-CoV-2 fatality measures such as Case Fatality Rate (CFR) and Infection fatality Rate (IFR). The CFR is typically defined as the number of deaths from COVID-19 as a proportion of the number of people who tested positive for the virus, whereas IFR is defined as the proportion of death among all infected people. CFR is often used to estimate the IFR, since the true extent of infection in a population and actual fatalities caused by this infection are often hard to establish. In the initial phase of the pandemic the presence of undetected infections proved to be a significant source of uncertainty for these observed fatality rates. In particular, the undetected asymptomatic cases lead to a substantial underestimation of the true case numbers. An additional level of uncertainty arises from the attribution problem relating to deaths. The classification of COVID-19 deaths varies by country. In countries such as the UK, deaths following a positive COVID-19 diagnosis are recorded as COVID-19 deaths, even if SARS-CoV-2 was not the direct cause, potentially leading to misattribution due to the absence of a prior diagnosis [34, 35]. This attribution problem increases with undetected asymptomatic infections.

Given the testing rate *γ*, the true CFR at time *t* is defined as ${CFR}_{true}=\frac{\int_{0}^{t}{{[d}^{s}I^{s}(\sigma )+d}^{a}I^{a}(\sigma )+d^{T}T(\sigma )]d\sigma }{\int_{0}^{t}\gamma [{p^{s}I}^{s}(\sigma )+{p^{s}I}^{a}(\sigma )]d\sigma }$, which obtains when no differences in T&I compliance exist between the two infective groups–that is when they share the same probability of testing, *p*^*s*^. The reported CFR, however, depends on actual diagnostic probability *p*^*a*^(*t*) and the number of deaths among the undiagnosed asymptomatics, *d*^*a*^*I*^*a*^(*t*), that and can be represented as ${CFR}_{rep}=\frac{\int_{0}^{t}\left[d^{s}I^{s}(\sigma )+{\theta d}^{a}{I^{a}(\sigma )+d}^{T}T(\sigma )\right]d\sigma }{\int_{0}^{t}\gamma {[p^{s}I}^{s}(\sigma )+{p^{a}(\sigma )I}^{a}(\sigma )]d\sigma }$, where *θ*∈[0,1] is the fraction of deaths among the undiagnosed asymptomatics that are eventually attributed to COVID-19. At low rates of infection prevalence, *p*^*a*^(*t*) is low, although the detected COVID-19 deaths are likely to be high. Thus, *CFR*_*rep*_ is likely to exceed *CFR*_*true*_. Eventually, *CFR*_*rep*_ decreases with rising prevalence rate as *p*^*a*^ rises in response. At high prevalence rates, *CFR*_*rep*_ is potentially lower than *CFR*_*true*_ if the detection rate exceeds the rate of undetected deaths, that is *p*^*a*^>*θd*^*a*^. Fig A in S2 File illustrates the extreme cases of *θ* = 0 and *θ* = 1. Similar uncertainties and biases afflict the IFR measure. In the absence of population-wide serological testing to determine prior infection status, these fatality measures are rendered unreliable. Consequently, any policy decisions based on these numbers would be flawed.

### 4.1 Implications for epidemic management policy

The behavioral model has two major policy implications. First, growth of disease transmission in this model is intrinsically self-limiting. The self-limiting mechanism arises because higher infection prevalence increases T&I behavior among the asymptomatics, reducing the pool of infectives, and preventing sharp spikes in infection. Second, the containment of a disease becomes increasingly more challenging with declining disease prevalence and falling likelihood of T&I. This is critical for containment policies that are primarily based on T&I behavior. Similar inferences can be drawn from vaccine-based prevention policies when the demand for vaccine is prevalence-dependent [12].

The behavioral model offers critical insights for restrictive epidemic management policies, such as lockdown. Interestingly, the optimality of stringency of lockdown depends on whether stringency is determined by the effective reproduction number, or by the simpler measures such as infection and/or fatality rates. To elucidate the differences in policy biases, let us analyze a social planner problem who can lockdown a fraction *π*(*t*)∈[0, 1] of the population. First, let the stringency of lockdown, *φ*_*t*_∈[0, 1], depends positively on the effective reproduction rate–i.e., $\varphi_{t}=\varphi (R_{t}^{b})$, *φ*′>0. Individuals are assumed to die only from infection. In the absence of a lockdown, each individual produces *y* units of output. If $*V* denotes the value of a statistical life, the planner should decide to lockdown if discounted cost of output foregone due to lockdown at the desired level of stringency is less than the discounted value of lives lost in absence of it–i.e., if $\int_{0}^{\infty }e^{-rt}\{y\varphi (R_{t}^{b})\pi (t)[S(t)+I^{s}(t)+I^{a}(t)]-\$VD(t)\}dt<0$, where *r*>0 is the planner’s discount rate. Since *p*^*a*^ is overestimated and the magnitude of $R_{t}^{b}$ underestimated at low values of infection, the desired stringency level $\varphi (R_{t}^{b})$ is likely to be *underestimated* in the initial and late phases of the pandemic. Hence, given everything else, the cost of death from less stringent lockdown is likely to be suboptimally high at low levels of prevalence.

On the other hand, if the level of stringency is determined by the detected infection rate *T*(*t*)/*S*(*t*) that significantly discounts the number of silent infections, the level of required stringency *φ*(*T*(*t*)/*S*(*t*)) would always be *underestimated* in the initial and late phases of the pandemic when *p*^*a*^ is low. This would imply a completely orthogonal pandemic management policy relative to the case above.

Given the policy dilemma regarding lockdown stringency stems from the uncertainty around *p*^*a*^, it is worthwhile to discuss the utility of non-pharmaceutical policies such as increasing (i) testing capacity (testing rate, *γ*) and (ii) testing compliance through a targeted public health campaign that increases the responsiveness of *p*^*a*^ to observed infection rate, *μ*. Increasing testing capacity improves access to tests, which is equivalent to a reduction in private cost of testing (*c*_*T*_ in section 3.1)–for example, through increasing the number of community testing facilities, development and better provisioning of accurate and rapid self-test kits. On the other hand, a public health campaign run by educating those infected on the benefits of testing could improve compliance with T&I protocols. This approach not only promotes testing but also helps to reduce the psychological costs associated with self-isolation, as detailed in section 3. In what follows, a parameterized model is simulated to explore the differential predictions of the behavioral SLIITReD model and a baseline ‘standard’ model that incorporates asymptomatic infections but assumes exogenous T&I behavior. Next, the simulated model analyzes the relative epidemiological effects of the two policies that relate to increasing testing capacity and testing compliance.

## 5. Predictions of the behavioral model

The parameter values reported in the literature are marked by substantial variation in demographics, pandemic phases, and depend on non-standardized definitions of detection rate, fatality rate, infectious period, etc. Numerical determination of these parameters is a significant challenge, given that an infinite number of different parameter sets could match the data equally well [36]. However, the main purpose of this section is not to provide quantitatively accurate predictions of the SLIITReD model, but instead offer an analytical comparison of the epidemiological outcomes between the standard and the behavioral model and depict the policy implications of the latter.

The infection transmission rate *β* is assigned a value of 0.9 to generate a peak infection at around the 60-day mark from the onset of the pandemic. The value for *κ* (transition rate from latency to infectious state) is 0.2, reflecting a latency period of 5 days [37]. The value of *ϕ* (proportion of symptomatic infections) is chosen to be 0.6 following the current estimates based on a representative sample [6]. Baccini et al. [37] estimated a median period of 4 days from symptoms to test, which implies a testing rate *γ* = 0.25. The magnitude of *δ* (transmission factor for asymptomatic infections) is not well-estimated in the literature. Using Italian data Giordano et al. [36] suggested an effective magnitude (= *βδ* in this model) for asymptomatic transmission of 0.57 on day 1 of the outbreak, implying a maximum magnitude of 0.63. A value *δ* = 0.6 is chosen. The values of *r*^*s*^ and *r*^*T*^ are based on the estimated 28-day time interval from symptoms to recovery for the undetected symptomatics (i.e., *r*^*s*^ = 1/28) and a 24-day interval from test to recovery (i.e., *r*^*T*^ = 1/24). Using German data Grimm et al. [38] estimated a recovery time for asymptomatic infections of 10 days, implying *r*^*a*^ = 0.10. The death rate *d*^*T*^ (death rate among the diagnosed) is assigned a value of 0.01 as in Giordano et al. [36]. The death rates *d*^*s*^ and *d*^*a*^ for the undetected symptomatics and asymptomatics, respectively, are both assigned a value of 0.01 following Park et al. [39].

Finally, the free scaling factor *μ* in *p*^*a*^ is assumed to be 0.2, implying an average magnitude of *p*^*a*^ of 4.15 percent, which is much higher than the best available estimate [37]. The probability of testing for the symptomatics is assumed to be 0.5 –the upper limit reported in Contreras et al. [26], which generates a daily effective testing rate of 12.5 percent among the symptomatic population. These parameters are identical in the benchmark ‘standard’ model that identifies the two infective groups as well as the ‘Test/Isolated’ compartment (for comparability) but assumes a probability of undertaking T&I among the asymptomatics (*p*^*a*^ = 0.04) that equals the mean value of *p*^*a*^ in the SLIITReD model.

**Fig 2:**
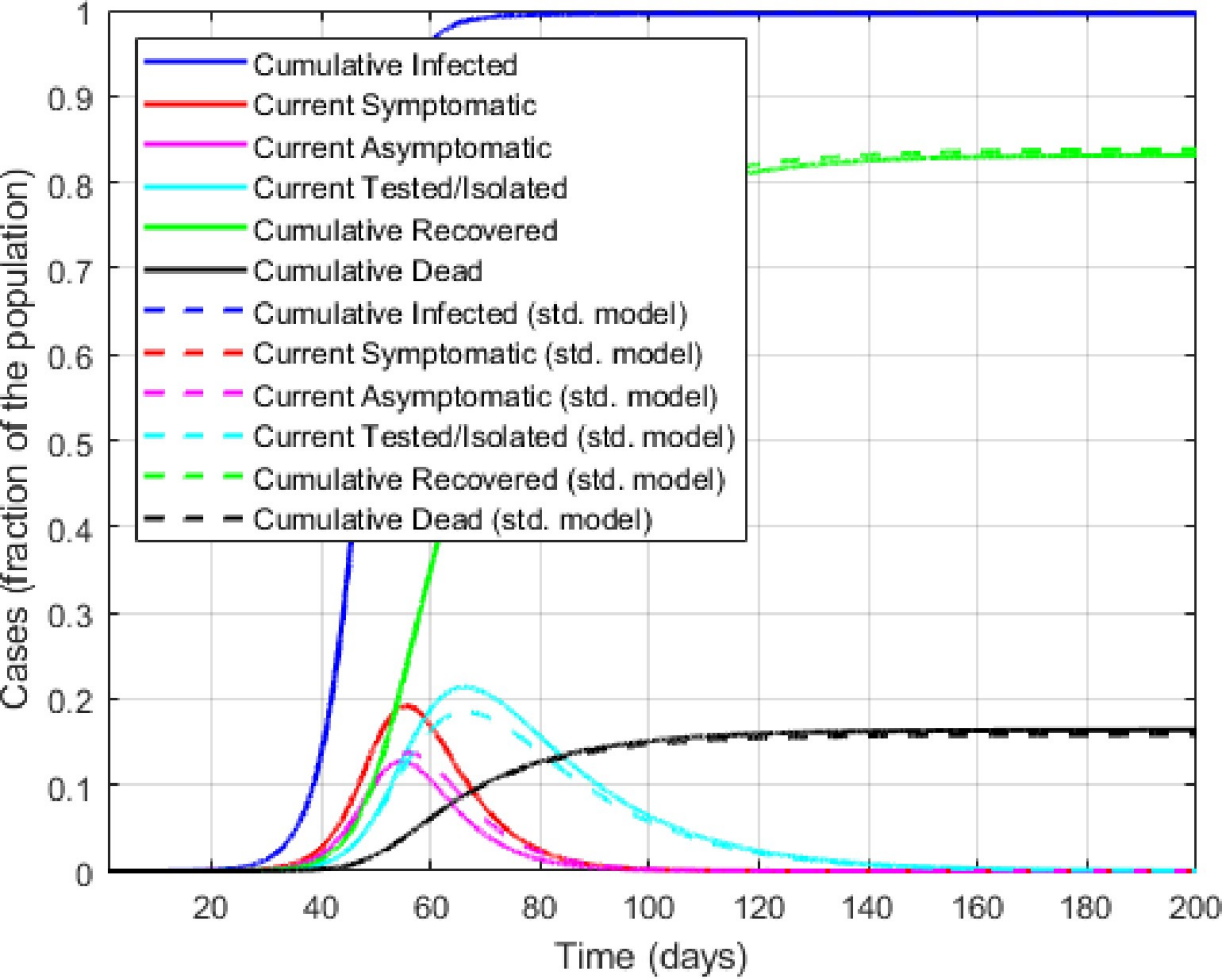
Qualitative model predictions of the SLIITEReD model versus the standard model with exogenous behavior.

Fig 2 illustrates the evolution of some key epidemiological measures over a 200-day horizon in the SLIITReD model vis-à-vis the standard model. Given the above parameterization, all differences between the two models should be attributed to the temporal difference in the asymptomatic T&I behavior. As conjectured, the standard model underpredicts this behavior, especially as observed infections rise. Prevalence dependent behavior thus flattens the peak in detected cases as the asymptomatic ramps up their T&I behavior in response to increasing infection rate. Because of the undetected infections, the standard model in Fig 2 grossly underpredicts the death rate and over-predicts the recovery rate in equilibrium. In both model 99 percent of the population is infected at the end of the horizon. Fig 2 shows that the behavioral model generates a higher mortality rate of about 16.4 percent and a recovery rate of approximately 83.1 percent. In the standard model, these death and recovery rates are 15.9 percent and 83.7 percent, respectively. Note that, conditional on 99 percent of the population being infected, underestimation of true CFR translates to a quantitatively similar underestimation of true IFR.

The implied value of the basic reproduction number ($R_{0}^{b}$) is 5.12, falling to its lowest value of 0.04 at around the 100^th^ day. The probability *p*^*a*^ also follows the infection curves with a lag, attaining a maximum value of 0.06 attained around day 65, before levelling out to 0 at around day 160. While the magnitudes of these differences are sensitive to the chosen parameter values, the basic insight is clear: the presence of undetected infections and failure to identify adaptive T&I behavior underestimate both the COVID-19 fatality rate and the duration of infection prevalence.

**Fig 3:**
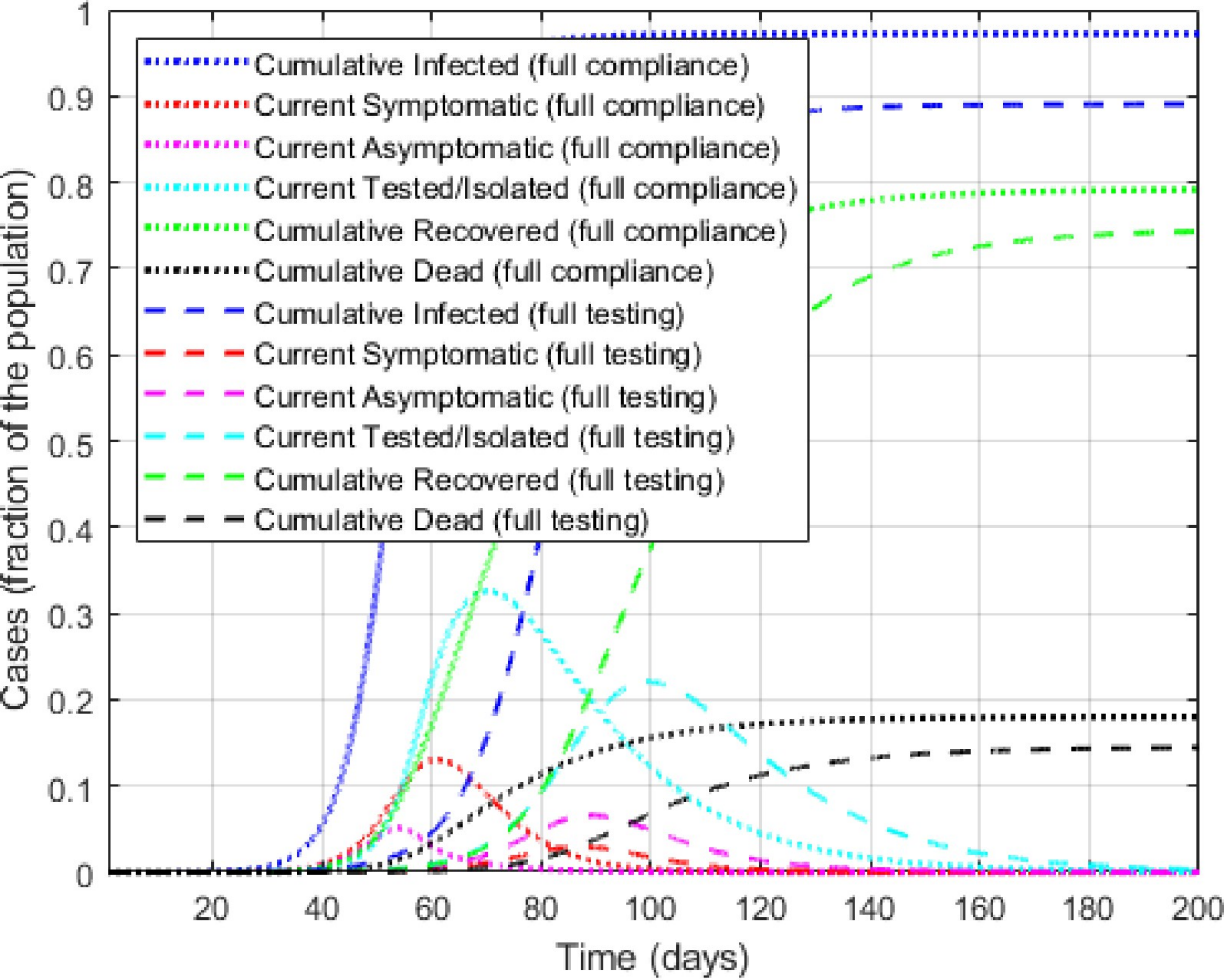
Behavioral model predictions for policies with (i) full test compliance and (ii) full testing capacity.

Next, let us examine the policy impacts of increasing (i) testing capacity or testing rate, *γ* and (ii) testing compliance, e.g., through a targeted public health campaign. Since both policies aim to increase the detection of the asymptomatic cases, the infection dynamic, recovery and death rates, as expected, depend on the magnitudes of the recovery and fatality rates among the asymptomatic cases relative to those of the diagnosed. For example, greater diagnostic efforts would lower the overall death rate only if the fatality rate among the undiagnosed is higher than among the diagnosed, which is true for the chosen parameter values. Hence, the policy impacts of increasing testing compliance and testing capacity should promote testing, increase the overall recovery rate, and reduce the overall mortality rate. We explore the effects of these two competing policy impacts in Fig 3.

Fig 3 compares the hypothetical scenarios under two alternative policies: a public awareness campaign that increases the probability of testing among the asymptomatics to the level of the symptomatics, and a mass testing campaign that achieves the maximum testing capacity. The awareness campaign increases the value of *μ* in *p*^*a*^ from 0.2 to 6.26 so that the *mean* of *p*^*a*^(*t*) = $\bar{p^{a}}=p^{s}$ = 0.5, the upper limit of symptom-driven testing rate [26]. This intervention is targeted to the asymptomatic because the symptomatics already have the maximum incentive to test. Under the full testing policy, the magnitude of *γ* that reflects the testing rate, increases from 0.25 to 1.

First, a comparison between Figs 2 and 3 makes it clear that relative to the baseline, both policies would ‘flatten’ the infection curves through increased testing. Counterintuitively, these interventions also lower cumulative recovery rates and increase fatality rates. This is due to two reasons. First, the overall infection rate is lower under both policies, resulting in fewer recoveries. Second, the undetected asymptomatics have a higher recovery rate (0.10) than those who test and isolate (0.042). Thus, increased testing mechanically slows down recovery for the asymptomatics, which drives the fatality rate up in this compartmental model.

A comparison between the two policies in Fig 3 reveals that under the full testing policy the infection curves are much flatter and long-tailed compared to awareness campaign policy. This is because the latter increases testing only among the asymptomatics, while the former increases access to tests thereby allowing greater testing for both infective groups, effectively spreading the testing over a longer period. The overall infection rate is much lower under the full testing policy, which results in lower recovery and fatality rates. Clearly, boosting test capacity and isolating the infected is key to flattening the infection curve, reducing disease spread, and lowering mortality, making it the preferred policy for disease control. Such prescription is also echoed in the literature [40, 41].

The predictive validity of the behavioral model was tested by recalibrating the parameters and replicating real infection data from Italy, as shown in Table A in S2 File. The trajectories for the current numbers of ‘recovered’, ‘dead’, ‘total infected’, and the ‘number of asymptomatic cases’ predicted by the simulated SLITReD model are compared with the corresponding values from the official data for the first 46 days. As depicted in Fig B in S2 File, the dynamics of these variables are all well-replicated by the SLIITReD model. The biases in the CFR due to unascertained COVID-19 deaths, as observed in the Italian case, are depicted in Fig C in S2 File.

## 6. Discussion

Considerable uncertainty surrounded the extent of local transmission of SARS-CoV-2 at the onset of the pandemic globally. We now know that the main source of this uncertainty was the undetected transmissions [2]. Although research on COVID-19 has informed us of the clinical nature of the virus and the effects of its spread, we still lack a coherent picture of the disease-behavior interaction and how the latter played a role in the dissemination of the virus.

The epidemiological model presented in this paper sheds light on the early spread of COVID-19, particularly the role of asymptomatic carriers and their T&I behaviors. It suggests that without incentives for T&I, asymptomatic individuals inadvertently accelerated the virus’s spread, especially when cases were undetected [42, 43]. This issue was pronounced in the United States and Europe, where it led to a surge in cases and deaths. The United States saw a resurgence of infections after initial containment measures waned [19]. The model also finds that higher infection rates improve the detection of asymptomatic cases, as evidenced by the relationship between test positivity and reported prevalence [31].

The study offers crucial insights for policy and preparedness for future pandemics. It reveals that undetected infections lead to an underestimation of the initial disease burden, suggesting that actual infection rates may surpass those reported. The model demonstrates that unattributed infections and deaths distort critical measures of disease severity, which can influence the implementation of restrictive health policies like lockdowns. Moreover, a vast proportion of ‘excess deaths’–the number of deaths during a specific period that exceed the expected number of deaths under normal conditions–is directly attributable to undetected SARS-CoV-2 infection [44]. Unattributed deaths contribute to an underestimation of excess mortality, with lower testing capacities exacerbating this issue. Msemburi et al. [45] estimated that total excess deaths during 2020 and 2021 were 2.4 to 3.1 times higher than reported COVID-19 deaths, indicating that asymptomatic fatalities likely comprised a significant portion of the observed ‘excess deaths’ during the pandemic’s initial wave.

The findings also underscore the necessity of allocating resources to enhance testing capacity, making diagnostic tests more accessible. Davis et al. [43] found that as of 8 March 2020, only 1–3 out of every 100 SARS-CoV-2 infections were detected in the United States and Europe, due to limited testing capacity. The results also align with the literature that prescribes urgent investment in a robust global COVID-19 monitoring system, potentially leveraging the infrastructure established for worldwide influenza surveillance. The development of a swift and reliable tracing-and-testing apparatus is vital, as others have advocated [26, 40, 41]. Right strategies to boost testing capacity even under limited resources can eventually prevent thousands of fatalities [46]. The study also suggests that awareness campaigns emphasizing the benefits of testing and isolation are beneficial during the early transmission phase. In general, resources should be directed towards efficiently identifying and isolating individuals capable of spreading SARS-CoV-2, rather than merely detecting the virus among those most likely to be infected, as observed in early 2020.

The study acknowledges limitations, such as the use of a deterministic epidemiological model that assumes homogenous and predictable T&I behavior among asymptomatic individuals, not accounting for variations due to factors like risk attitude, perception of self-efficacy, and pro-social attitude. The model also simplifies by assuming perfect compliance with self-isolation guidelines, combining T&I decisions into one. A more nuanced model could consider non-adherence to isolation guidelines as a separate decision influenced by behavioral factors. Such a model would likely show heightened peaks of infection and death if most asymptomatic individuals underestimate their potential to spread the disease. Additionally, the infection dynamics could be enriched by incorporating other aspects, such as the possibility of reinfection after recovery, vaccination decisions leading to reduced risk, etc., temporary immunity through infection and vaccination, etc. These considerations present potential avenues for future research.

## Supporting information

S1 File Supplementary materials. (DOCX)

S2 File Supplementary materials. (DOCX)
