# Supplementary material for: To test or not to test? A new behavioral epidemiology framework for COVID-19
Source: PLoS One. 2024 Dec 17;19(12):e0309423. doi: 10.1371/journal.pone.0309423 (PMC11651578; doi:10.1371/journal.pone.0309423)
Supplement: S1 File — (DOCX) [file pone.0309423.s001.docx]

**S1. Supporting information:** Derivations of Reproduction Numbers; SLITReD Model with Imperfect Isolation & Reinfection Possibility

**1.1 *Derivation of the Basic Reproduction Number:***

To understand the system behavior, we partition it into 3 subsystems: The first includes just variable $S$ (corresponding to susceptible individuals), the second includes $L$, $I^{s}, I^{a}$, $T$, (the exposed and infected individuals), which are non-zero only during the transient, and the third includes variables $R$ and $D$ (representing recovered and dead). We focus on the second subsystem, which we denote the $[L,I^{s},I^{a},T]$ subsystem.

Variables $R$ and $D$ (which are monotonically increasing) converge to their asymptotic values $\bar{R}$and $\bar{D}$, and $S$ (which is monotonically decreasing) converges to $\bar{S}$ *iff:* $\lim_{t\to\infty} L\left( t \right)=\lim_{t\to\infty} I^{s}\left( t \right)=\lim_{t\to\infty} I^{a}\left( t \right)=\lim_{t\to\infty} T\left( t \right)=0$.

Define $x={[L I^{s}I^{a} T]}^{T}$ as the number of individuals in each ‘infected and/or isolated’ compartment. Assume that a Disease-free equilibrium $x_{0}$ = $\left\{ 0, 0, 0, 0 \right\}$ exists and is stable in the absence of the disease. We can rewrite the *L-I^s^-I^a^ -T* subsystem in the linearized form $\frac{dx(i)}{dt}=\mathcal{F}_{i}\left( x \right)-\mathcal{V}_{i}\left( x \right)$ for $i=1,\ldots,4$, where $\mathcal{F}_{i}\left( x \right)$ is the rate of appearance of new infections in compartment 𝑖 and $\mathcal{V}_{i}\left( x \right)$ is the rate of other transitions between compartment 𝑖 and other infected compartments.

Define $F=\left[ \frac{\partial\mathcal{F}_{i}\left( x_{0} \right)}{\partial x_{j}} \right]$ and $V=\left[ \frac{\partial\mathcal{V}_{i}\left( x_{0} \right)}{\partial x_{j}} \right]$ for $i\geq1$ and $j\leq4$.

Biologically, *F* is entry-wise non-negative and *V* is a non-singular *M*-matrix, so $V^{-1}$ is entry-wise nonnegative. The basic reproduction number is given by: $R_{0}=\rho({FV}^{-1})$, where 𝜌 denotes the spectral radius. Matrix ${FV}^{-1}$ has $(i,j)$ entry equal to the expected number of secondary infections in compartment 𝑖 produced by an infected individual introduced in compartment 𝑗.

In our $[L I^{s} I^{a} T]$ sub-model around the initial numbers ${S=S}_{0}$,$I^{s}=I^{a}=R=D=0, T=T_{0}, p^{a}\left( 0 \right)$:

$F=\left[ \begin{matrix} 0 & \beta S_{0} & \beta{\delta S}_{0} & 0 \\ 0 & 0 & 0 & 0 \\ 0 & 0 & 0 & 0 \\ 0 & 0 & 0 & 0 \end{matrix} \right]$ ;

$$V=\left[ \begin{matrix} \kappa& 0 & 0 & 0 \\ -\phi\kappa& \gamma p^{s}+{r^{s}+d}^{s} & 0 & 0 \\ -(1-\phi)\kappa& 0 & {\gamma p^{a}\left( 0 \right)+r}^{a}+d^{a} & 0 \\ 0 & 0 & 0 & r^{T}+d^{T} \end{matrix} \right]$$

yielding, ${FV}^{-1}=\left[ \begin{matrix} \frac{\beta\phi S_{0}}{{\gamma p}^{s}+r^{s}+d^{s}}+\frac{\beta(1-\phi)\delta S_{0}}{\gamma p^{a}\left( 0 \right)+{r^{a}+d}^{a}} & \frac{\beta S_{0}}{{\gamma p}^{s}+r^{s}+d^{s}} & \frac{\beta\delta S_{0}}{{{\gamma p^{a}\left( 0 \right)+r}^{a}+d}^{a}} & 0 \\ 0 & 0 & 0 & 0 \\ 0 & 0 & 0 & 0 \\ 0 & 0 & 0 & 0 \end{matrix} \right]$

Thus, the behavioral *R_0_* is given by:

$$R_{0}^{b}=\frac{\beta S_{0}\left\{ \delta\left( 1-\phi\right)\left( \gamma p^{s}+r^{s}+d^{s} \right)+\phi\left( \gamma p^{a}\left( 0 \right)+{r^{a}+d}^{a} \right) \right\}}{\left( {\gamma p}^{s}+r^{s}+d^{s} \right)\left( \gamma p^{a}\left( 0 \right)+{r^{a}+d}^{a} \right)}$$

**1.2.** ***Derivation of the Effective Reproduction Number:***

Rewriting the $[L I^{s} I^{a} T]$ sub-model around the current numbers $S(t)$, $I^{s}(t)$, $I^{a}\left( t \right)$, $p^{a}\left( t \right)$, $T\left( t \right)$, $R(t)$ and $D(t)$, and denoting $\aleph\left( t \right)\equiv S\left( t \right)+T\left( t \right)+R\left( t \right)+D(t)$, the *M*-matrix is given by:

$$M=\left[ \begin{matrix} \kappa& 0 & 0 & 0 \\ -\phi\kappa& \gamma p^{s}+{r^{s}+d}^{s} & 0 & 0 \\ -(1-\phi)\kappa& 0 & {\frac{\gamma\mu T(t)}{\aleph\left( t \right)}+r}^{a}+d^{a} & \left( \frac{\gamma\mu}{\aleph\left( t \right)} \right)\left( 1-\frac{T(t)}{\aleph\left( t \right)} \right)I^{a}\left( t \right) \\ 0 & 0 & 0 & r^{T}+d^{T} \end{matrix} \right]$$

Denoting $\psi^{s}={r^{s}+d}^{s}$, $\psi^{a}={r^{a}+d}^{a}$ and $\psi^{T}={r^{T}+d}^{T}$, we get:

$${FV}^{-1}=\left[ \begin{matrix} \frac{\beta\phi S(t)}{{\gamma p}^{s}+\psi^{s}}+\frac{\beta(1-\phi)\delta S(t)\aleph\left( t \right)}{\gamma\mu T(t)+\psi^{a} \aleph\left( t \right)} & \frac{\beta S(t)}{{\gamma p}^{s}+\psi^{s}} & \frac{\beta\delta S(t)\aleph\left( t \right)}{\gamma\mu T(t)+\psi^{a} \aleph\left( t \right)} & -\frac{\beta S\left( t \right)}{\psi^{T}}\left( 1+\frac{\gamma\mu\delta[\aleph\left( t \right)-T\left( t \right)]I^{a}\left( t \right)}{\gamma\mu T(t)+\psi^{a} \aleph\left( t \right)} \right) \\ 0 & 0 & 0 & 0 \\ 0 & 0 & 0 & 0 \\ 0 & 0 & 0 & 0 \end{matrix} \right]$$

The eigenvalue of this ${FV}^{-1}$ matrix yields the expression in equation (5):

$$R_{t}^{b}=\beta S(t)\left( \frac{\phi}{\gamma p^{s}+\psi^{s}}+\frac{\delta\left( 1-\phi\right)}{\gamma\mu\left\{ T\left( t \right)/\aleph\left( t \right) \right\}+\psi^{a}} \right)$$

**1.3 The SLITReD Model with Imperfect Self-isolation**

This is an augmented SLITReD model that includes a separate decision to isolate for the asymptomatics. Let a fraction $\sigma$ of asymptomatic individuals test but do not self-isolate $(0<\sigma<1)$. For stability of the model, we require that $\sigma\leq\gamma$ – that is, the rate at which people avoid isolation must not exceed the rate at which they get tested. The asymptomatic individuals who avoid self-isolation continue in the $I^{a}$ compartment and continue to infect the susceptibles on contact.

Let the individuals who tested positive for COVID-19 infection, decide to self-isolate at the rate of $\omega$. This is the rate at which people exit the test (*T*) compartment and go into the *Q* (self-isolation) compartment. People in the *Q* compartment move to recovery (*R*)and dead (*D*) compartments at the rates of $r^{Q}$ and $d^{Q}$, respectively. The dynamic system of this expanded model can then be represented as:

$S^{'}=-\beta\left\{ I^{s}\left( t \right)+{\delta I}^{a}\left( t \right) \right\}S\left( t \right)$ (5a’)

$L^{'}=\beta\left\{ I^{s}\left( t \right)+{\delta I}^{a}\left( t \right) \right\}S\left( t \right)-\kappa L(t)$ (5b’)

${I^{s}}^{'}=\phi\kappa L\left( t \right)-\left\{ {\gamma p}^{s}+{r^{s}+d}^{s} \right\}I^{s}\left( t \right)$ (5c’)

${I^{a}}^{'}=\left( 1-\phi\right)\kappa L\left( t \right)-\left\{ {(\gamma-\sigma)p}^{a}\left( t \right)+{r^{a}+d}^{a} \right\}I^{a}\left( t \right)$ (5d’)

$T^{'}={\gamma p^{s}I}^{s}\left( t \right)+{\gamma p}^{a}\left( t \right)I^{a}\left( t \right)-\omega T\left( t \right)-\sigma{\gamma p}^{a}\left( t \right)I^{a}\left( t \right)$ (5e’)

$Q^{'}=\omega T\left( t \right)-\left( r^{Q}+d^{Q} \right)Q\left( t \right)$ (5f’)

$R^{'}=r^{s}I^{s}\left( t \right)+{r^{a}I}^{a}\left( t \right)+r^{Q}Q\left( t \right)$ (5g’)

$D^{'}=d^{s}I^{s}\left( t \right)+{d^{a}I}^{a}\left( t \right)+d^{Q}Q\left( t \right)$ (5h’)

$p^{a}\left( t \right)=\frac{\mu T\left( t \right)}{S\left( t \right)+T\left( t \right)+R\left( t \right)+D(t)}$ (5i’)

Note that equation (5d’) now includes those asymptomatics who refuse to quarantine, equation (5e’) is now reflects the number of such asymptomatics, and equation (5f’) is the new equation that corresponds to the self-isolation state.

As opposed to the main model, this augmented model has five infected states, {*L, I^s^, I^a^, T, Q*}. Taking this sub-system through the process described above, we get:

$$F_{Q}=\left[ \begin{matrix} 0 & \beta S_{0} & \beta{\delta S}_{0} & 0 & 0 \\ 0 & 0 & 0 & 0 & 0 \\ 0 & 0 & 0 & 0 & 0 \\ 0 & 0 & 0 & 0 & 0 \\ 0 & 0 & 0 & 0 & 0 \end{matrix} \right]$$

$$V_{Q}=\left[ \begin{matrix} \kappa& 0 & 0 & 0 & 0 \\ -\phi\kappa& \gamma p^{s}+{r^{s}+d}^{s} & 0 & 0 & 0 \\ -(1-\phi)\kappa& 0 & ({\gamma-\sigma)p^{a}\left( 0 \right)+r}^{a}+d^{a} & 0 & 0 \\ 0 & -\gamma p^{s} & -(\gamma-\sigma)p^{a}\left( 0 \right) & \omega& 0 \\ 0 & 0 & 0 & -\omega& r^{Q}+d^{Q} \end{matrix} \right]$$

yielding, ${F_{Q}V_{Q}}^{-1}=\left[ \begin{matrix} \frac{\beta\phi S_{0}}{{\gamma p}^{s}+r^{s}+d^{s}}+\frac{\beta(1-\phi)\delta S_{0}}{(\gamma-\sigma)p^{a}\left( 0 \right)+{r^{a}+d}^{a}} & \frac{\beta S_{0}}{{\gamma p}^{s}+r^{s}+d^{s}} & \frac{\beta\delta S_{0}}{{{(\gamma-\sigma)p^{a}\left( 0 \right)+r}^{a}+d}^{a}} & 0 & 0 \\ 0 & 0 & 0 & 0 & 0 \\ 0 & 0 & 0 & 0 & 0 \\ 0 & 0 & 0 & 0 & 0 \\ 0 & 0 & 0 & 0 & 0 \end{matrix} \right]$

The resulting behavioral *R_0_* is given by:

$$R_{0,Q}^{b}=\frac{\beta S_{0}\left\{ \delta\left( 1-\phi\right)\left( \gamma p^{s}+r^{s}+d^{s} \right)+\phi\left( (\gamma-\sigma)p^{a}\left( 0 \right)+{r^{a}+d}^{a} \right) \right\}}{\left( {\gamma p}^{s}+r^{s}+d^{s} \right)\left( (\gamma-\sigma)p^{a}\left( 0 \right)+{r^{a}+d}^{a} \right)}$$

Note that $R_{0,Q}^{b}$ is identical to the original $R_{0}^{b}$, except that $\gamma p^{a}\left( 0 \right)$ has been replaced by $(\gamma-\sigma)p^{a}\left( 0 \right)$ in both the numerator and the denominator. Hence, addition of the self-isolation compartment, making isolation imperfect among the infected asymptotic individuals, does not change the qualitative nature of the dynamic of the disease; it only reinforces the effects of testing decision. The higher the magnitude of $\sigma$, the higher is the impact of $(\gamma-\sigma)$ on $R_{0,Q}^{b}$. Higher $R_{0,Q}^{b}$ results in a higher scale of infection. This is not surprising, because some infectious people who fail to self-isolate, will continue to spread the disease, the infection curve will peak a little higher and a little later, depending on the value chosen for $\omega$.

The SLITReD model omits the *Q* compartment assuming when people test, they already internalize the cost of isolation which effectively reduces the probability of testing.

**1.4 Inclusion of Reinfection Possibility in the Model:**

Here I derive $R_{0}$ including reinfection possibilities in the model. That is, immunity from the disease following recovery is temporary, and a fraction of people who recovered become susceptible again. This situation is examined using a simple Susceptible – Latent - Infected – Recovered – Susceptible (SLIRS) model as described below. The objective is to show that the basic reproduction rate is identical to that of the SLIR model.

A simple dynamic system for the SLIRS model is represented by the following ODEs:

$S^{'}=-\beta I(t)S\left( t \right)+\omega R(t)$ (a)

$L^{'}=\beta I(t)S\left( t \right)-\kappa L(t)$ (b)

$I^{'}=\kappa L(t)-\gamma I(t)$ (c)

$R^{'}=\gamma I\left( t \right)-\omega R(t)$ (d)

The new term *ωR* in equation (d) denotes the number of recovered individuals who experience a protective period of 1/ω and subsequently lose immunity and become susceptible again. These individuals return to the *S* compartment – as shown by the addition of *ωR* to equation (a).

The basic reproduction rate can be computed from equations (b) and (c) by setting $L^{'}+I^{'}>0$ and $S\left( t \right)$ = $S_{0}$. Thus, when $L^{'}+I^{'}>0$, we have $\beta I\left( t \right)S_{0}-\gamma I\left( t \right)>0$, or $R_{0}>\frac{\gamma}{\beta S_{0}}=\frac{\gamma}{\beta}$ for $S_{0}=1$, i.e., when almost everybody in the population is susceptible. Note that the presence of the reinfection does not affect $R_{0}$. This is because the infection dynamics is driven by what is happening in the transient compartments, $L(t)$ and $I\left( t \right)$ here.

To see how reinfection of the recovered is neutralized in a constant population, rewrite (a) as $\beta I\left( t \right)S\left( t \right)= \omega R\left( t \right)-S^{'}$, and rewrite (d) as $\gamma I\left( t \right)=R^{'}+\omega R\left( t \right)$. Now, $L^{'}+I^{'}=\beta I\left( t \right)S\left( t \right)-\gamma I\left( t \right).$Substituting, we have $L^{'}+I^{'}=\omega R-S^{'}-\left( R^{'}+\omega R \right)=-{(S}^{'}+R^{'})$. Because ${(S}^{'}+R^{'})$ remains unchanged by the introduction of *ωR*, the infection dynamic remains unchanged. Thus, reinfection of the recovered does not affect $R_{0}$ when population is constant (the last equation amounts to $L^{'}+I^{'}+S^{'}+R^{'}=0$). This is also true for the behavioral model.
