# Supplementary material for: To test or not to test? A new behavioral epidemiology framework for COVID-19
Source: PLoS One. 2024 Dec 17;19(12):e0309423. doi: 10.1371/journal.pone.0309423 (PMC11651578; doi:10.1371/journal.pone.0309423)
Supplement: S2 File — (DOCX) [file pone.0309423.s002.docx]

**S2. Supporting information**

Fig A: The evolution of actual and reported measures of CFR in the SLIITReD model

Table A: Initial values of the SLIITReD model parameters

|  | **Epidemiological meaning** | **Value** | **Guiding source** |
| --- | --- | --- | --- |
| $\beta$ | Transmission rate | 0.44 | Assumed |
| $\kappa$ | Transition rate from latency to infectious state | 0.22 | Baccini et al. [37] |
| $\phi$ | Proportion of symptomatic infections | 0.66 | Assumed |
| $\gamma$ | Diagnosis rate | 0.18 | Baccini et al. [37] |
| $\delta$ | Transmission factor for asymptomatic infections | 1.11 | Assumed |
| $p^{s}$ | Probability of diagnosis for symptomatic infections | 0.50 | Contreras et al. [26] |
| $r^{s}$ | Recovery rate for symptomatic infections | 0.01 | Grimm et al. [38] |
| $r^{a}$ | Recovery rate for asymptomatic infections | 0.01 | Assumed |
| $r^{T}$ | Recovery rate among the self-isolated | 0.01 | Giordano et al. [36] |
| $d^{s}$ | Death rate for symptomatic infections | 0.015 | Baccini et al., [37] |
| $d^{a}$ | Death rate for asymptomatic infections | 0.01 | Baccini et al., [37] |
| $d^{T}$ | Death rate among the self-isolated | 0.01 | Giordano et al., [36] |
| $\mu$ | Scaling parameter in $p^{a}$ | 0.75 | Assumed |

Table A presents the parameter values that are inferred from the official data on the evolution of the epidemic in Italy from 20 February 2020 (day 1) through 5 April 2020 (day 46), available at Protezione Civile as reported in the dataset in Giordano et al. [36]. The data horizon (46 days) is chosen to match the work in Giordano et al. [36], which provides a reliable benchmark.

As in this study, the parameters are updated based on successive public health measures implemented by the policymakers. The fraction of the population in each stage at day 1 is set as: L(1) = 300/60e06, $I^{s}$(1) = 180/60e6, $I^{a}$=120/60e06, T(1) = 20/60e06, R(1) = D(1) = 0; $S = 1 - L -$ $I^{s}$ – $I^{a}$ $- R - T$ $- D$.

After day 4, basic social-distancing measures were implemented and consequently, the public became aware of the epidemic outbreak and of the basic hygiene recommendations (such as frequent hand washing, avoiding handshakes and keeping distance) and early school closures by the Italian government, we set $\beta$ = 0.43.

After day 12, a policy came into effect that limited screening to symptomatics only, which decreased the transmission rate to $\beta$ = 0.415 and the probability of testing by the symptomatics from 0.5 to 0.55. People who were completely asymptomatic, did not change their probability of testing.

After day 22, as a result of the partial lockdown $\beta$ = 0.395 and increased the probability of testing by the asymptomatics in response to the rising detected incidence rate.

After day 28, the lockdown became fully operational and stricter (e.g., going out for work was no longer allowed, all non-indispensable activities were stopped gradually). Thus, we set $\beta$ = 0.28, $\delta$ = 0.66, $\gamma$ = 0.34, and $d^{T}$ = 0.0012.

After day 38, a wider testing campaign was launched. The parameter values changed to $\beta$ = 0.23, $\delta$ = 0.29, $\gamma$ = 0.42, and $\mu$ = 0.79.

The SLIITReD model is simulated using the parameters described in Table A. The behavior of the epidemiological variables predicted by the simulated model are compared with the corresponding values from the official data for the first 46 days are depicted in Fig B. The trajectories for the current numbers of ‘recovered’, ‘dead’, ‘total infected’, and the ‘number of asymptomatic cases’ are all well-replicated by the SLIITReD model. The simulated model assumes that all deaths of the undiagnosed infected individuals, regardless of their symptom status, are counted in the COVID-19 death tally. Similarly, ‘recovered’ include all recoveries from COVID-19 among symptomatics and asymptomatics. Unlike Giordano et al. (2020), the SLIITReD model reproduces the disease fatality rate well, as shown in Fig B.

Fig B: Replication of Italian data using the simulated SLIITReD model

The simulations presented in Giordano et al (2020) were unable to replicate the data on COVID-19 deaths that corresponds to $D(t)$ in the SLIITReD model – the observed data appeared particularly high with respect to the CFR reported in the literature. In the current simulation, the model is able to reproduce fatality figures. As argued by Giordano et al. (2020), the corresponding CFR is high due largely to the high proportion of older people (50 years and above) in the Italian population and the steep age-gradient of CFR reported across all countries, and by the extensive intergenerational contacts in the Italian society, which propelled the virus transmission from younger to the older and more fragile generations.

The high CFR can also be explained by an overestimation of COVID-19 fatalities in Italy since the official numbers for COVID-19 deaths provisionally included the deaths of all people tested positive for the SARS-CoV-2, even when they had multiple pre-existing comorbidities and the exact cause of death had not yet been ascertained.

While statistical distortion due to provisional data is a challenge in calibrating the model to initial data, in particular with respect to the ratio of fatality to detected cases (CFR), which could be overestimated due to typical over-ascertainment of COVID-19 deaths in the initial phases of a pandemic. However, as discussed in the main text, CFR could also be high due to the lower number of detected cases resulting from test avoidance behavior among the asymptomatics when infection prevalence is low.

Fig C: Predicted biases in the COVID-19 fatality measures in Italy

The SLIITReD model reproduced the death figures without substantial fine-tuning of the parameters. Indeed, Fig C shows that the reported CFR, when all unascertained deaths were excluded from the fatality count, was higher than the true CFR in the initial phase of the pandemic in Italy. As more cases were detected, the reported CFR converged to the true CFR in the middle phase and fell below the true CFR thereafter, as predicted. Of course, the reported CFR depends on the proportion of deaths among the undetected asymptomatics that are ascertained as COVID-19 deaths. Fig C depicts that when all COVID-19 deaths are ascertained correctly, the reported CFR exceeded the true CFR throughout the period. The kink on both curves on day 28 resulted from significant changes in the policy measures described above.

The purpose of this replication exercise is not to establish the predictive ability of the model for practice, but simply to show that the model could be a useful guidance to build more accurate predictive models that integrates testing and isolation behavior and the feedback between prevalence and behavior that affects the course of the pandemic itself. The current model has a few caveats as some of the technical assumptions may not be valid in practice. For example, the model implicitly assumes that transition times from one compartment to the next follows an exponential distribution, which may not be true in data. In practice, the time spent in a compartment is affected not only by the epidemiological characteristics of the virus but also heterogenous social, demographic, and economic characteristics across locales and regions affecting disease propagation (Baccini, 2021).
